# Polyethylene Biodegradation by an Artificial Bacterial Consortium: *Rhodococcus* as a Competitive Plastisphere Species

**DOI:** 10.1264/jsme2.ME24031

**Published:** 2024-07-31

**Authors:** Jyothi Priya Putcha, Wataru Kitagawa

**Affiliations:** 1 Graduate School of Agriculture, Hokkaido University, Kita-9 Nishi-9, Kita-ku, Sapporo 060–8589, Japan; 2 Bioproduction Research Institute, National Institute of Advanced Industrial and Technology (AIST), 2–17–2–1, Tsukisamu-Higashi, Toyohira Ward, Sapporo 062–8517, Japan

**Keywords:** biodegradation, polyethylene, consortium, *Rhodococcus*, plastisphere

## Abstract

Polyethylene (PE), a widely used recalcitrant synthetic polymer, is a major global pollutant. PE has very low biodegradability due to its rigid C-C backbone and high hydrophobicity. Although microorganisms have been suggested to possess PE-degrading enzymes, our understanding of the PE biodegradation process and its overall applicability is still lacking. In the present study, we used an artificial bacterial consortium for PE biodegradation to compensate for the enzyme availability and metabolic capabilities of individual bacterial strains. Consortium members were selected based on available literature and preliminary screening for PE-degrading enzymes, including laccases, lipases, esterases, and alkane hydroxylases. PE pellets were incubated with the consortium for 200 days. A next-generation sequencing ana­lysis of the consortium community of the culture broth and on the PE pellet identified *Rhodococcus* as the dominant bacteria. Among the *Rhodococcus* strains in the consortium, *Rhodococcus erythropolis* was predominant. Scanning electron microscopy (SEM) revealed multilayered biofilms with bacteria embedded on the PE surface. SEM micrographs of PE pellets after biofilm removal showed bacterial pitting and surface deterioration. Multicellular biofilm structures and surface biodeterioration were observed in an incubation of PE pellets with *R. erythropolis* alone. The present study demonstrated that PE may be biodegraded by an artificially constructed bacterial consortium, in which *R. erythropolis* has emerged as an important player. The results showing the robust colonization of hydrophobic PE by *R. erythropolis* and that it naturally possesses and extracellularly expresses several target enzymes suggest its potential as a host for further improved PE biodeterioration by genetic engineering technology using a well-studied host-vector system.

Plastics are high-mole­cular-weight synthetic polymers derived from petrochemicals (Ahmed *et al.*, 2018). The versatility and low cost of plastics have established them as integral components in virtually every industry. However, the extensive use of plastics, particularly single-use plastics, has led to a significant increase in the amount of global solid waste. Approximately 9% of the estimated 6,300 Mt of global plastic waste generated between 1950 and 2015 was recycled and 12% was incinerated; the remaining 79% ended up in landfills or the environment (Geyer *et al.*, 2017). Current approaches for the disposal and management of plastic waste are not sustainable. Therefore, research efforts towards the development of eco-friendly polymers, such as biopolymers, and the microbial degradation of plastics are rapidly gaining momentum. Microorganisms harboring synthetic polymer-degrading enzymes are regarded as green tools for recycling plastics. The recently discovered bacterium *Ideonella sakaiensis* was found to produce two key enzymes that break down poly(ethylene terephthalate) (PET) into its monomeric forms (Yoshida *et al.*, 2016).

Polyethylene (PE) is the most widely used and produced plastic polymer and its biodegradation presents unique challenges (Geyer *et al.*, 2017). The microbial biodegradation of plastics begins with their colonization by microorganisms, which establish a “plastisphere” community, with the term plastisphere referring to a diverse community of microorganisms present on the surface of plastics (Zettler *et al.*, 2013; Zhang *et al.*, 2022). However, microbial colonization poses a significant challenge because of the hydrophobic nature of PE. Microorganisms capable of producing metabolites, such as biosurfactants, or forming biofilms to overcome this hydrophobicity have potential as plastisphere inhabitants (Pathak and Navneet, 2017; Zhang *et al.*, 2022). Furthermore, PE has additional limitations due to its high mole­cular weight and carbon (C-C) backbone. These factors significantly contribute to its high recalcitrance to biodegradation.

Chemical and biochemical processes that reduce mole­cular weights and induce polymer oxidation are crucial for making PE more accessible to microorganisms. These processes are often achieved by the synergistic effects of abiotic (photo/thermal oxidation) and biotic factors (such as biosurfactants and exoenzymes) (Restrepo-Flórez *et al.*, 2014). The microbial colonization of PE initiates the biodegradation process, followed by the enzymatic breakdown of the polymer, which involves both extracellular and intracellular enzymes. Extracellular enzymes are crucial for the depolymerization of PE via hydrolytic cleavage to generate smaller oligomers. These oligomers are then taken up by microbial cells for further metabolism facilitated by intracellular enzymes (Cai *et al.*, 2023).

To date, the enzymes reported for PE degradation are generally associated with lignin or hydrocarbon degradation. Lignin-degrading enzymes, such as laccases, manganese peroxidases, and lignin peroxidases, and hydrocarbon-degrading enzymes, including the AlkB family of alkane hydroxylases (AHs), have been implicated in the degradation of PE. Both enzyme classes play a role in the depolymerization of PE, with AHs being involved in terminal and subterminal oxidation (Wei and Zimmermann, 2017). Additionally, hydrolases, such as lipases and esterases, are surface-modifying enzymes that increase the surface hydrophilicity of plastic polymers, promoting microbial colonization (Cai *et al.*, 2023). The current literature postulates the role of enzymes, such as laccases and a few peroxidases, in the depolymerization of the PE polymer. A mole­cular-docking study hypothesized a mechanism involving a PE-laccase reaction, in which the active site of laccase interacts with PE and participates in a series of electron transfer reactions. As a result, more oxygen-containing functional groups (such as alcohols and aldehydes) are introduced into the PE molecule, eventually resulting in depolymerization. The smaller oligomers generated are amenable for further metabolism. Additionally, enzymes including AHs oxidize *n*-alkanes into their corresponding alcohols and subsequently into their corresponding carboxylic acids, which are further metabolized by the *β*-oxidation pathway. In a similar manner, the subterminal hydroxylation of alkanes produces esters, which are further hydrolyzed by esterases, eventually entering the *β*-oxidation pathway (Ghatge *et al.*, 2020; Santacruz-Juarez *et al.*, 2021; Jin *et al.*, 2023). The collective effects of these enzymes facilitates the hydrolytic cleavage of PE polymers/oligomers, ultimately leading to the mineralization of PE (Ghatge *et al.*, 2020; Amobonye *et al.*, 2021). A few bacterial and fungal species that express these enzymes, such as *Rhodococcus ruber* and *Phanerochaete chrysosporium*, have been shown to degrade PE to some extent (Iiyoshi *et al.*, 1998; Gilan *et al.*, 2004). Despite these findings, the practicality of using microbes and their corresponding genes and enzymes for PE biodegradation is severely lacking, and our understanding of the underlying mechanisms remains incomplete. Moreover, we have yet to isolate microorganisms that are capable of fully degrading and mineralizing pristine PE.

Individual bacterial strains may have limited metabolic capabilities; therefore, they may not be fully effective in breaking down PE when used alone. Effective PE breakdown and utilization by a single microorganism has yet to be reported. One approach to address this limitation is the construction and application of a microbial consortium comprising a specialist microbe for each enzyme. Another approach is to genetically engineer a suitable host that expresses the target PE-degrading enzymes.

In the present study, we investigated the former strategy, in which we constructed and evaluated the performance of an artificial bacterial consortium for degrading PE. We assembled a consortium comprising bacterial strains belonging to genera or species previously associated with PE degradation and assessed the activity of various PE-specific enzymes, including laccases, lipases, esterases, and AHs, with respect to their extracellular expression. Select bacterial strains were assembled into a consortium to utilize their combined potential for PE biodegradation. Additionally, we aimed to examine and select a suitable consortium strain for use as a versatile host in a genetic engineering strategy. We assessed consortium strains based on their colonization capability, long-term survival on PE, and their possession of PE degradation-related enzyme genes in order to select the most effective strain as the candidate host.

## Materials and Methods

### Chemicals and media

Guaiacol, Tween-20, and PE (SIGMA 428043-250G; low density, pellet form, diameter 3–4‍ ‍mm) were purchased from Sigma-Aldrich. *n*-Hexadecane and *n*-eicosane were purchased from Wako Pure Chemical Industries, *n*-dotriacontane from TCI Chemicals, and Tween-80 from MP Biomedicals. Luria–Bertani (LB) and W-minimal media (Kitagawa *et al.*, 2018) were used as cultivation media. Their specific compositions were as follows: LB medium (L^–1^ of medium): 10‍ ‍g tryptone, 5‍ ‍g yeast extract, 5‍ ‍g NaCl, and 15‍ ‍g agar; W-minimal medium: 1,000× buffer stock solution (L^–1^ of medium): 10.75‍ ‍g MgO, 2‍ ‍g CaCO_3_, 4.5‍ ‍g FeSO_4_·7H_2_O, 1.44‍ ‍g ZnSO_4_·7H_2_O, 1.12‍ ‍g MnSO_4_·4H_2_O, 0.25‍ ‍g CuSO_4_·5H_2_O, 0.28‍ ‍g CuSO_4_·7H_2_O, 0.06‍ ‍g H_3_BO_3_, and 51.3‍ ‍mL conc. HCl; 100× buffer stock solution (1 L): 10‍ ‍g MgSO_4_·7H_2_O, 0.5‍ ‍g FeSO_4_·7H_2_O, and 100‍ ‍mL 1,000× buffer; 1× buffer (990 mL): 0.85‍ ‍g KH_2_PO_4_, 4.9‍ ‍g Na_2_HPO_4_, and 0.5‍ ‍g (NH_4_)_2_SO_4_; final W-minimal medium (1 L): 990‍ ‍mL 1× buffer and 10‍ ‍mL 100× buffer.

### Microbe selection

To include the necessary PE-degrading enzymes, bacteria for the consortium were selected based on the literature on microbial PE degradation. To date, several bacteria have been reported to exhibit PE degradation activities, including *Rhodococcus* (Bonhomme *et al.*, 2003; Gilan *et al.*, 2004; Sauvageau *et al.*, 2009; Laczi *et al.*, 2015; Pi *et al.*, 2017; Gibu *et al.*, 2019; Zampolli *et al.*, 2022), *Brevibacillus* (Hadad *et al.*, 2005), *Enterobacter* (Yang *et al.*, 2014), *Bacillus* (Yao *et al.*, 2022), *Phormidium* (Sarmah and Rout,‍ ‍2018), *Hyphomonas* (Zettler *et al.*, 2013), *Alcanivorax* (Delacuvellerie *et al.*, 2019), *Pseudomonas* (Mukherjee *et al.*, 2018), and *Streptomyces* (El-Shafei *et al.*, 1998). Based on these studies and the availability of genome information, the same strain or closely related members from the reported literature were selected. Strains used in the present study are listed in Table 1 and reference strains in Supplemental Table S1. An *E. coli* strain was also included. *E. coli* was previously reported to form biofilms on plastic and, thus, was used as the enzyme assay control (Ganesan *et al.*, 2022).

### Extracellular enzyme assay and alkane utilization assay on agar plates

The following four enzymes are important for microbial PE degradation: laccases, esterases, lipases, and AHs (Restrepo-Flórez *et al.*, 2014; Ghatge *et al.*, 2020). Plate assays were performed to establish whether the investigated strains exhibited PE degradation/utilization enzyme activities, particularly extracellular activities. Among the strains listed in Table 1, two *Phormidium* strains were not used for the plate assay because they did not grow on solid agar media.

### Laccase plate assay

LB agar plates supplemented with 0.02% guaiacol and 0.5 or 1‍ ‍mM CuSO_4_ were used to screen for laccase activity. Bacterial strains were inoculated by streaking or spotting and were incubated at 28 or 37°C. The oxidation of guaiacol by laccase is indicated by the formation of a reddish-brown product. Extracellular laccase activity is observed as a reddish-brown halo around a bacterial colony, whereas intracellular laccase activity is present when the cells alone turn reddish-brown (Kiiskinen *et al.*, 2004).

### Lipase/esterase plate assay

Tween-20 and Tween-80 agar plates were used for this assay. They were prepared as follows: (L^–1^); 10‍ ‍g peptone, 5‍ ‍g NaCl, 0.1‍ ‍g CaCl_2_·2H_2_O, 20‍ ‍g agar, and 10‍ ‍mL (v/v) Tween-20/-80 (Kumar *et al.*, 2012). Bacterial strains were spotted onto these plates and incubated at 28 or 37°C. Tween-80 was used to detect lipase activity because it is primarily hydrolyzed by lipases due to its oleic acid esters, while Tween-20 was used to detect esterase activity because it contains esters of short-chain fatty acids. Lipase and esterase activities were indicated by the appearance of a calcium salt precipitate and/or the clearing of such a precipitate resulting from the complete degradation of the salt of the fatty acid around the bacterial colony (Sierra, 1957; Kumar *et al.*, 2012).

### Alkane utilization plate assay

Alkanes of varying carbon chain lengths were employed to assess alkane utilization by the bacterial strains selected. Specifically, *n*-hexadecane (C16, oil phase at an ambient temperature), *n*-eicosane (C20, solid phase), and *n*-dotriacontane (C32, solid phase) were used in the present study. Alkane test plates were prepared as follows. Regarding *n*-hexadecane, a 0.2% volume of the substrate was added to W-minimal medium agar and solidified. Concerning *n*-eicosane and *n*-dotriacontane, the substrates were initially dissolved in diethyl ether at a concentration of 5‍ ‍mg mL^–1^, and the liquid was then sprayed onto the surface of solidified W-minimal media agar (approximately 1‍ ‍mL plate^–1^). In this strategy, a thin layer of *n*-eicosane or *n*-dotriacontane formed on the plates. After the complete evaporation of the solvent, the plates were inoculated with bacteria. Control plates were prepared without alkane supplementation. Bacterial strains were spotted onto these plates and incubated at 28 or 37°C. Alkane utilization activity was indicated by the growth of the test strain on alkane-containing plates, while extracellular enzyme activity was evaluated by the formation of a clear zone around the colonies on *n*-eicosane- and *n*-dotriacontane-supplemented plates.

### Consortium construction and cultivation

Liquid hydrocarbons, such as *n*-hexadecane, have been reported to improve the accessibility of PE to bacteria when added to culture broth (Montazer *et al.*, 2020). Moreover, a study by Gilan *et al.* found that the addition of mineral oil to culture medium improved bacterial colonization and PE biodegradation (Gilan *et al.*, 2004). Therefore, *n*-hexadecane was added to the culture broth used in the present study. All the consortium strains were initially pre-cultured individually in W-minimal medium containing 0.5% hexadecane (*Bacillus subtilis* and *Streptomyces griseus* were pre-cultured in LB because their growth in W-minimal medium was very slow). The pre-culture broth (OD_600_=~2–3) was used as the seed inocula at the following volumes: 100‍ ‍μL of very fast growers in W-minimal medium (*R. ruber* and *R. jostii* RHA1), 150‍ ‍μL of fast growers (*R. rhodochrous*, *R. zopfii*, and *R. erythropolis*), 500‍ ‍μL of the inocula of bacteria with intermediate growth (*Alcanivorax* strains and *Pseudomonas fluorescens*), and 1‍ ‍mL of slow growers (*Hyphomonas* strains, *Brevibacillus borstelensis*, *Enterobacter asburiae*, *B. subtilis*, *Escherichia coli* K-12, *S. griseus*, and *Phormidium* strains) (Table 1). Independent seeds were mixed and introduced into a final volume of 100‍ ‍mL of W-minimal medium broth supplemented with 0.1% *n*-hexadecane. PE pellets were treated in a 70% ethanol bath for 15‍ ‍min and dried before adding them to the consortium. Thirty PE pellets were added per flask. The consortium culture was performed in triplicate (three flasks: R1, R2, and R3), and a flask without any bacterial inoculum was used as the control. Flasks were maintained at 28°C on a rotary shaker at 90‍ ‍rpm. Additionally, after 60 days, half of the spent media in the culture flasks was replaced with fresh W medium.

### Sampling, DNA extraction, and next-generation sequencing (NGS) of 16S rRNA genes

Bacterial samples from the consortium culture broth were collected on days 1, 10, 25, 40, 60, and 120 for DNA extraction and a community ana­lysis. PE samples were collected for plastisphere DNA extraction on day 120. DNA was extracted using the conventional phenol-chloroform extraction method (Sambrook *et al.*, 1989). Using the extracted DNA as the template, the bacterial V4 hypervariable region of the small-subunit rRNA (SSU rRNA) gene was amplified for all samples (consortium culture broth and plastisphere) using the primers 515F (5′-GTGYCAGCMGCCGCGGTAA-3′) and 806R (5′-GGACTACHVGGGTWTCTAAT-3′). Amplicon PCR and follow-up index PCR were performed according to the Illumina 16S Metagenomic Sequencing Library Preparation Guide (Part # 15044223 Rev. B; Illumina). PCR was conducted using a KAPA HiFi HotStart Ready Mix (Kapa Biosystems). The final DNA libraries were prepared according to the manufacturer’s instructions and ran on an Illumina iSeq100 Sequencing System using the iSeq 100 i1 Reagent v2 (300-cycle) kit (Illumina), with the run set for paired-end sequencing.

### Community ana­lysis

A downstream data ana­lysis for the FASTQ output from the Illumina iSeq100 sequencing step was performed using Quantitative Insights into Microbial Ecology 2 (QIIME 2) version 2019.10 (Bolyen *et al.*, 2019). The DADA2 plugin of QIIME2 was used for‍ ‍denoising, merging, and chimera removal and to construct an‍ ‍amplicon sequence variant (ASV) table (Callahan *et al.*, 2016).‍ ‍Regarding sequence references and taxonomic annotations, Silva 138 SSURef NR99 was used to train the classifier before annotating the reads (Quast *et al.*, 2013). Subsequent ana­lyses were‍ ‍performed using the marker data profiling module of MicrobiomeAnalyst (a web-based platform) (Dhariwal *et al.*, 2017; Chong *et al.*, 2020; Lu *et al.*, 2023). In the Data Filtering step, the‍ ‍following filters were applied: in the low count filter, the minimum count was set to 10, with prevalence in samples set to 10%. In the Data Normalization step, total sum scaling (TSS) was applied. Taxonomy bar plots and pie charts of the culture consortium and PE plastisphere communities were visualized with MicrobiomeAnalyst.

### Scanning electron microscopy (SEM)

PE samples were collected on days 10, 60, 120, and 200 for SEM observations. PE samples with biofilms, PE samples after biofilm removal, and respective control samples were obtained for SEM visualization and imaging. To remove biofilms, PE samples were treated with a lysozyme solution (2‍ ‍mg mL^–1^) and proteinase K (final concentration, 1‍ ‍mg mL^–1^) in STE buffer (0.1 M NaCl, 10‍ ‍mM Tris-HCl, and 1‍ ‍mM EDTA, pH 8.0), followed by a treatment with 2% SDS at 37°C for 3–4 h. Samples were then washed thrice with warm, sterile distilled water. SEM samples were fixed with 2% (w/v) glutaraldehyde in 0.1 M sodium cacodylate buffer (pH 7.2) at room temperature for 1 h. Stepwise dehydration was performed using a graded ethanol series (50, 75, 90, 95, and 100%) for 15‍ ‍min each, followed by substitution with dehydrated tert-butyl alcohol. Samples were then freeze-dried using a VFD-21S t-BuOH freeze dryer (Vacuum Device). Prepared SEM samples were mounted on aluminum stubs using carbon paste (Pelco Colloidal Graphite, Ted Pella), coated with gold using a DII-29010SCTR Smart Coater, and then visualized and imaged using a JSM-6010PLUS/LV SEM at 15 kV.

## Results and Discussion

### Extracellular enzyme assays and alkane utilization assays on agar plates

#### Laccase plate assay

**Fig. 1.**
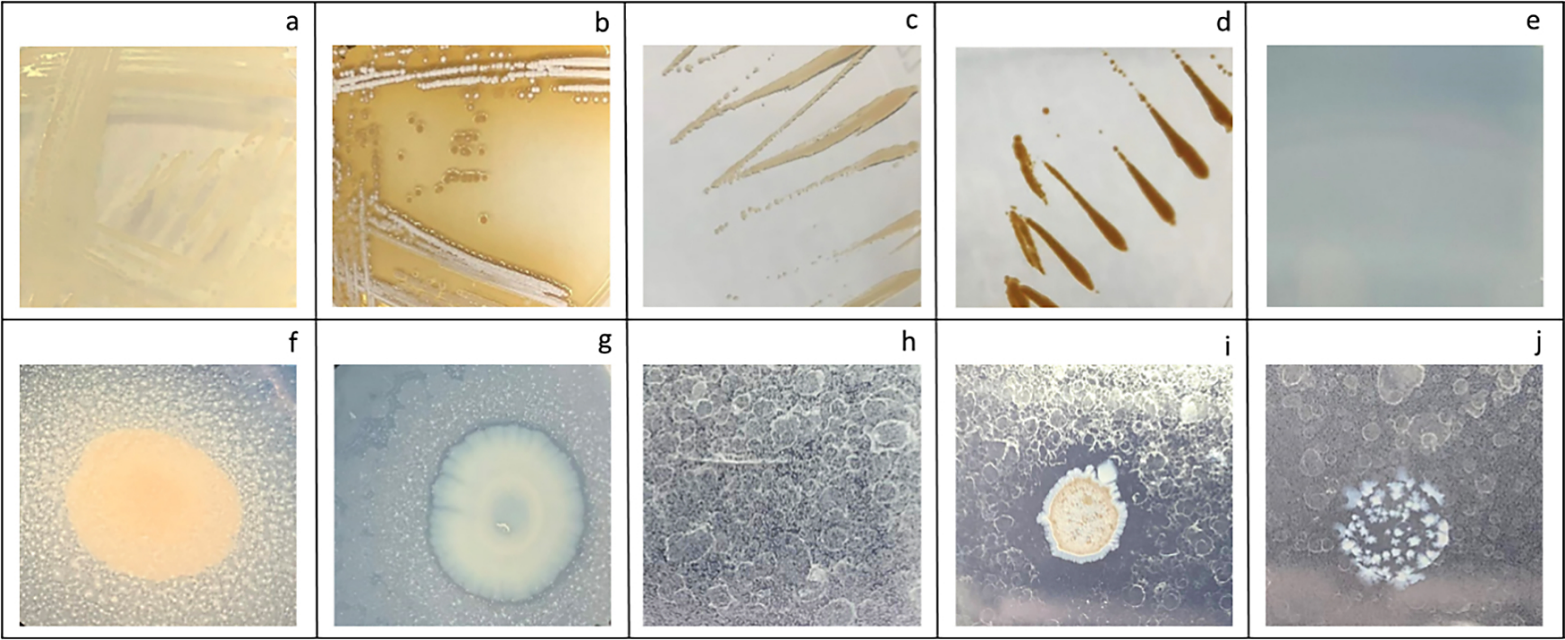
Plate assays. **Laccase plate assay (a, b, c, and d)**, (a) *Streptomyces griseus* on a control plate (without a substrate), (b) *S. griseus* on a test plate (with a substrate) exhibiting extracellular laccase activity, (c) *Hyphomonas polymorpha* on a control plate, (d) *H. polymorpha* on a test plate exhibiting intracellular laccase activity, **Esterase plate assay (e and f)**, (e) appearance of a Tween-20/-80 test plate without bacteria, (f) *Rhodococcus erythropolis* on a Tween-20 test plate showing precipitation (extracellular esterase activity), **Lipase plate assay:** (g) *Pseudomonas fluorescens* on a Tween-80 test plate showing precipitation (extracellular lipase activity), **Alkane utilization assay (h, i, and j)**, (h) *n*-eicosane-sprayed agar test plate showing surface-dried solid *n*-eicosane, (i) *Rhodococcus rhodochrous* on a test plate showing growth and a clear zone, (j) *Brevibacillus borstelensis* on a test plate showing growth.

Among the strains tested, only *S. griseus* displayed a reddish-brown halo around its colonies, indicating extracellular laccase activity (Fig. 1b). Most of the other strains displayed reddish-brown colonies with no halo, suggesting intracellular laccase activity, as observed for *Hyphomonas* strains (Fig. 1d) and *P. fluorescens* and *Rhodococcus* strains (Table 2). *B. subtilis* did not exhibit laccase activity. The two *Alcanivorax* strains did not grow on the test plates, which may have been due to their high sensitivity to copper, a requirement for laccase enzyme activity (Table 2). Based on the genomic information available, putative laccase genes were detected in all strains, with some possessing multiple genes. However, only intracellular laccase activity was detected in several strains. For example, *R. erythropolis* has seven copies of a putative laccase in its genome (Ausec *et al.*, 2011), and the presence of a signal peptide was predicted in all of them. Laccases are considered to play an important role in PE degradation by cleaving its backbone. Therefore, many microbial strains did not exhibit extracellular laccase activity, which is an important issue for their potential application. In contrast, the laccase gene *epoA* was previously reported in *S. griseus* (Endo *et al.*, 2002), the only species that exhibited extracellular activity. The PE degradation activity of *S. griseus* observed in this study was attributed to this enzyme.

### Esterase/lipase plate assay

Esterase activity was assessed using Tween-20. Nearly all of the strains tested displayed Ca salt precipitation, indicating positive esterase activity (Fig. 1e and f). *Brevibacillus borstelensis* and the two *Alcanivorax* strains displayed no growth, indicating their sensitivity to Tween-20. In the lipase assay, all tested strains produced Ca salt precipitates on Tween-80 agar plates, confirming their lipase activity (Fig. 1g and Table 2). These results indicate that extracellular esterase and lipase activities are ubiquitous, in contrast to laccase activity.

### Alkane utilization plate assay

All five *Rhodococcus* strains exhibited proper growth on all three *n*-alkanes (Table 2). *Hyphomonas polymorpha* and *E. asburiae* grew in *n*-hexadecane-containing media. Other strains showed either faint or no growth. Among these strains, *B. subtilis*, *E. coli*, and *S. griseus* grew on both control and alkane-supplemented agar plates, with slightly improved growth on alkane-supplemented agar plates. Therefore, these strains were marked as faint growth (fG) in Table 2. Since PE is a long alkane, microorganisms that assimilate long alkanes are advantageous for its decomposition. *Rhodococcus* was previously reported to utilize alkanes with a >C20 chain length in the liquid phase (Whyte *et al.*, 1998; Zampolli *et al.*, 2014). The present results confirmed the ability of *Rhodococcus* strains to grow on agar, indicating their potential for this application. Notably, only *Rhodococcus* strains produced clear zones on *n*-eicosane-supplemented agar plates (Fig. 1i). To the best of our knowledge, this is the first study to identify a microorganism that produces a clear zone on solidified *n*-eicosane. AlkB, a well-known AH that is also widely distributed among the genus *Rhodococcus*, is a membrane-integrated enzyme that requires an electron transport protein to function (Vomberg and Klinner, 2000; Takei *et al.*, 2008; Xiang *et al.*, 2023). Another known AH, CYP153 (cytochrome P450) is a cytoplasmic enzyme that also requires an electron transport protein (van Beilen and Funhoff, 2007; Ji *et al.*, 2013). Since these enzymes are membrane-bound or cytoplasmic, they are active intracellularly and, thus, unlikely to function outside the cell to form the clear zone. These rhodococci may also produce as yet unknown extracellular alkane-degrading enzymes. For example, extracellular AHs were previously reported to be produced by thermophilic bacteria, which are involved in crude oil degradation (Yusoff *et al.*, 2020). Alternatively, the biosurfactant produced increased alkane solubility, making it invisible on the plates. Nevertheless, both cases offer significant advantages for alkane and PE degradation.

The plate assays for enzymes, including laccases, esterases, lipases, and AHs, revealed that none of the individual bacterial strains expressed all four target enzymes extracellularly (Table 2). Moreover, the degree of enzyme production varied among the different bacterial strains. Therefore, the utilization of these strains as a consortium may compensate for these discrepancies, not only in terms of enzyme profiles, but also in the production of other beneficial metabolites, such as biosurfactants (Mukherjee *et al.*, 2016). Each strain expressed at least one of the target enzymes extracellularly; therefore, they were included in the consortium.

### Consortium culture with PE and a consortium ana­lysis

The majority of consortium studies have used microbial isolates from enriched or natural plastisphere sources. For example, Joshi *et al.* constructed a bacterial consortium with four bacterial isolates from marine plastic debris (Joshi *et al.*, 2022), Han *et al.* built a consortium of *Arthrobacter* sp. and *Streptomyces* sp. isolated from agricultural soils (Han *et al.*, 2020), and D’Souza *et al.* formulated a fungal consortium from pure cultures of three *Aspergillus* spp. (DSouza *et al.*, 2021) for PE degradation.

In the present study, we constructed an artificial bacterial consortium where all the constituent members were tested for enzymatic activity related to PE degradation, for the first time. The resulting bacterial consortium was employed for the biodegradation of PE.

### Culture broth community ana­lysis

**Fig. 2.**
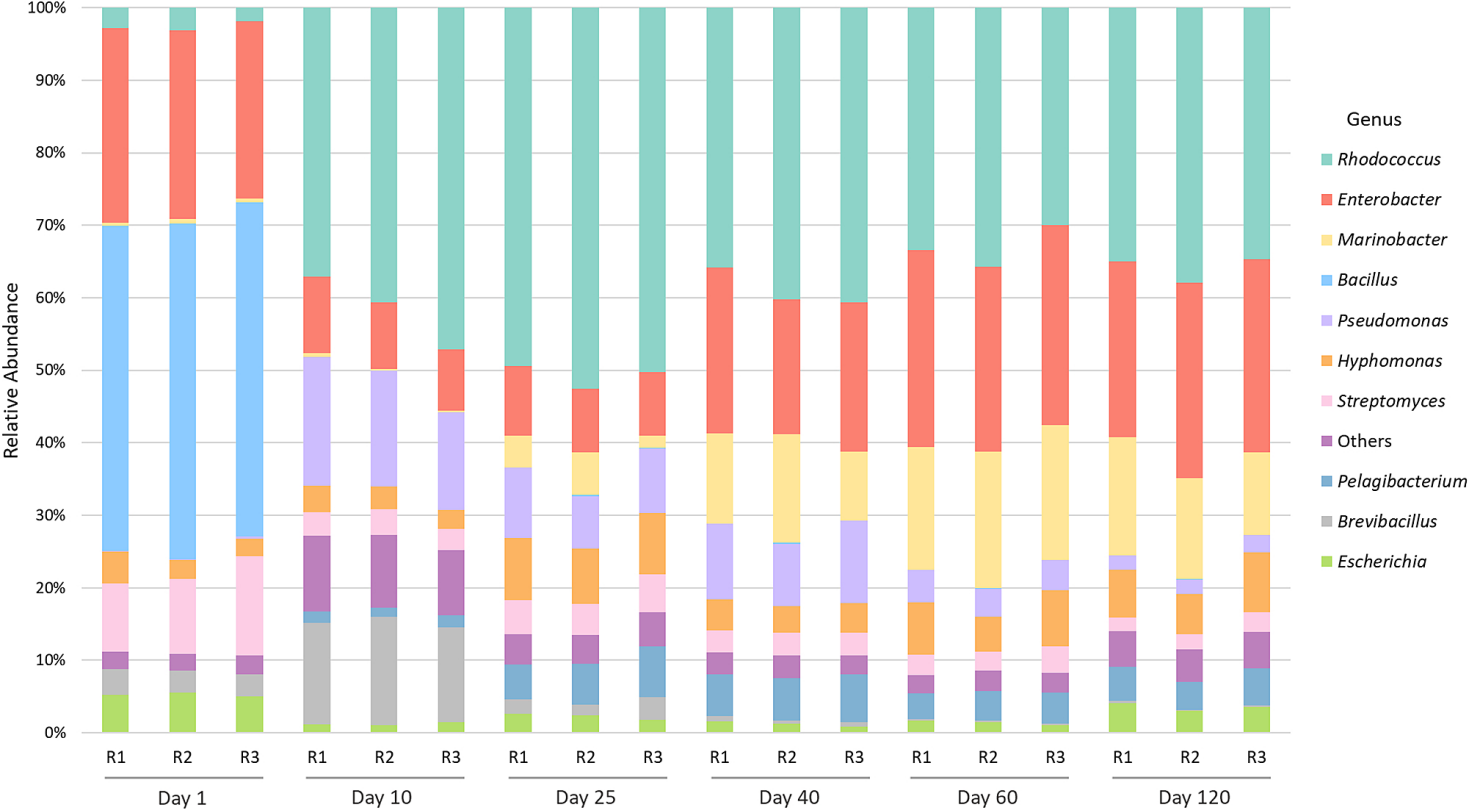
Taxonomy bar plots illustrating culture broth community dynamics over 120 days, showcasing the top 10 abundant genera on each sampling day (R1, R2, and R3 denote triplicate flasks).

The taxonomy bar plots shown in Fig. 2 depict the dynamics of the culture broth community over 120 days, displaying the top 10 abundant genera on each sampling day. The culture broth community markedly changed from days 1 to 10 and then gradually became a mostly stable community thereafter. A significant shift was noted in the relative abundance of consortium members in the first few days (days 1–10), which was characterized by the marked growth of *Rhodococcus* and a pronounced decrease in the abundance of *Bacillus*. Initial fluctuations due to microbe-microbe interactions (such as competition) subsided, and subsequently, the culture broth community gradually changed and reached a more stable state. The community ana­lysis, being DNA-based, may have also included DNA from dead cells; therefore, the stability of the consortium did not necessarily imply that all members were actively breaking down PE. In this context, consortium stability may be interpreted as a state in which these bacteria coexist, with some directly contributing to PE degradation and others merely playing a supportive role.

A stable community composition was evident from days 40 to 120. The dominant taxa observed after day 40 were *Rhodococcus*, *Enterobacter*, *Marinobacter*, and *Hyphomonas*.

Some bacteria, such as *Marinobacter* and *Pelagibacterium*, were detected in the culture broth, but were not part of the initial consortium community. This unexpected result may be attributed to non-axenic cultures of *Phormidium* strains harboring hydrocarbonoclastic contaminants that thrive under culture conditions. The association of these bacteria with *Phormidium* was confirmed via a 16S rRNA sequence ana­lysis of the original stock of two *Phormidium* cultures from a bioresource organization (data not shown). The culture collection note clearly stated that these stocks were a mixture of multiple strains with a strong symbiotic relationship; therefore, contamination did not change the outcomes of the experiment. The presence of *n*-hexadecane in the consortium culture may have facilitated the growth of these bacteria. It is important to note that *Pelagibacterium* is associated with oil-contaminated soils, while *Marinobacter* is a well-known hydrocarbonoclastic bacterium (Huang *et al.*, 2021). Furthermore, a study conducted by Sun *et al.* showed that *Marinobacter* strains exhibited robust growth with *n*-hexadecane as the sole carbon source. Additionally, these strains have been shown to express several AH genes (Sun *et al.*, 2018). *n*-Hexadecane, which was added at the start of the culture, was expected to be consumed quickly by *Rhodococcus* and other consortium bacteria. However, the continuous presence of these contaminant hydrocarbonoclastic bacteria over a long period may also have contributed to the decomposition of PE.

### Plastisphere community ana­lysis

**Fig. 3.**
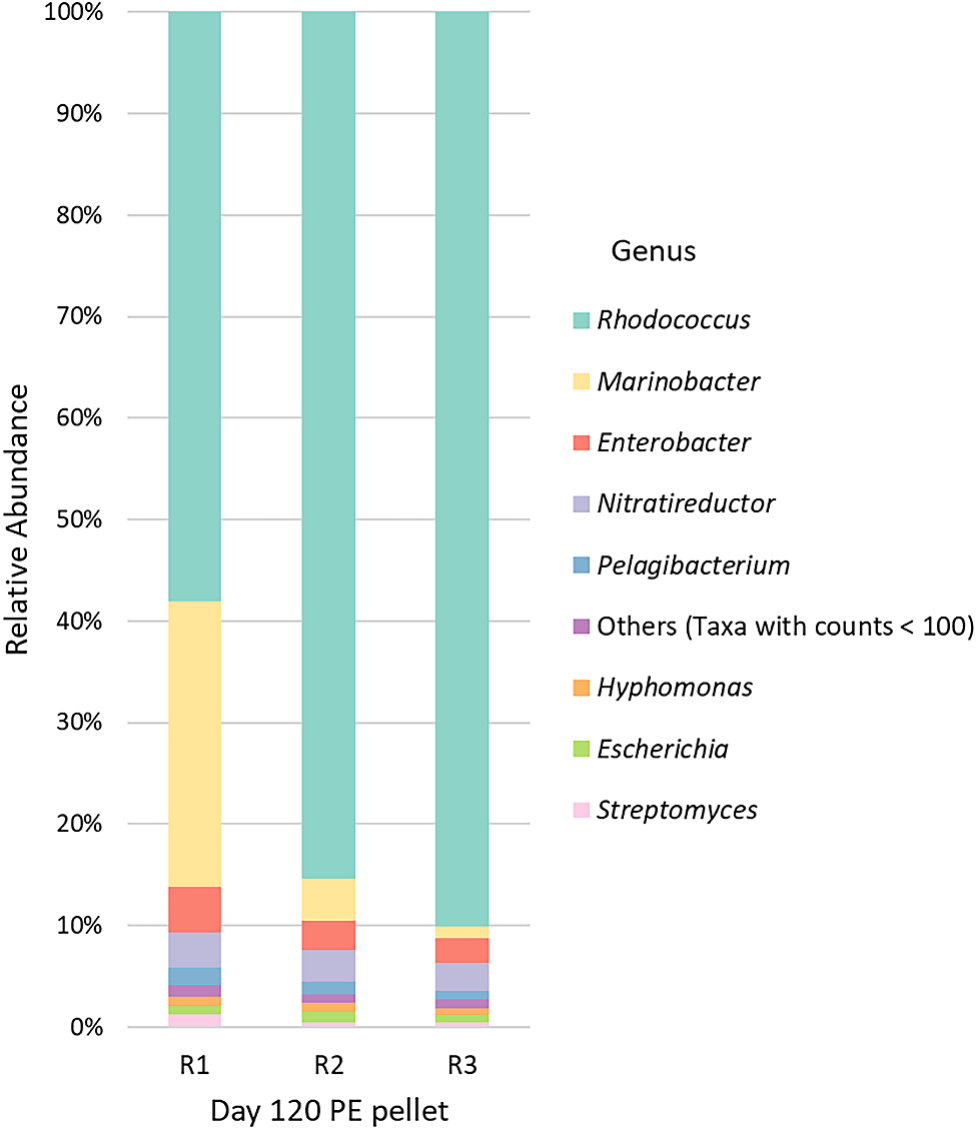
Taxonomy bar plots depicting the plastisphere community (Day 120) (R1, R2, and R3 denote triplicate flasks).

The taxonomy bar plots in Fig. 3 show the plastisphere communities in PE samples on day 120. In comparison with its relative abundance in the culture broth (36%), *Rhodococcus* dominated the plastisphere community, accounting for approximately 78% of bacteria (average value of triplicates; see also Supplemental Fig. S1). The high relative abundance of *Rhodococcus* in the plastisphere suggests its potential role as a primary colonizer that establishes itself early and‍ ‍produces robust long-term biofilms on the PE surface. Previous studies demonstrated that many established plastisphere microorganisms inherently produced biofilms; *Rhodococcus* naturally forms biofilms (Amaral-Zettler *et al.*, 2020; Pátek *et al.*, 2021). The presence of *Rhodococcus* in plastispheres has been reported: Wang *et al.* suggested the potential role of *R. erythropolis* as a microplastic degrader in a mangrove microplastic plastisphere study (Wang *et al.*, 2024), Ya *et al.* showed the enrichment of *Rhodococcus* in PE microplastics (Ya *et al.*, 2022), and Rüthi *et al.* identified *Rhodococcus* as a plastisphere bacterium in Alpine and Arctic soils (Ruthi *et al.*, 2020).

The early establishment of *Rhodococcus* may have enhanced its competitive advantage for colonizing PE pellets, potentially hindering the proliferation of other strains (Amaral-Zettler *et al.*, 2020). Additionally, *Rhodococcus* produces biosurfactants in response to hydrocarbons. The presence of *n*-hexadecane in the culture broth may have induced biosurfactant production in *Rhodococcus* strains, thereby improving colonization. Biosurfactants specifically localized on the outer cell surface of *Rhodococcus* cells increase the hydrophobicity of their cell surface and facilitate bacterial attachment to the hydrophobic PE surface (Cappelletti *et al.*, 2020). *Rhodococcus* sp. generally produce trehalose-based glycolipid biosurfactants (Cappelletti *et al.*, 2020). For example, *R. qingshengii* was found to carry genes involved in the production of trehalose-based glycolipid biosurfactants, such as *otsA*, *otsB*, *treY*, and *treZ* (Markova *et al.*, 2023). The genome information of *R. erythropolis* and *R. rhodochrous* used in the present study also revealed the presence of these biosurfactant genes.

*Rhodococcus* ASVs obtained from the NGS data ana­lysis were further exami­ned to distinguish the five *Rhodococcus* strains used in the consortium. We identified *R. erythropolis* as the sole prominent species within the entire *Rhodococcus* community, establishing its dominance in both the culture broth and plastisphere. *R. erythropolis* outperformed the other *Rhodococcus* strains in terms of the growth and colonization of PE. These results on *R. erythropolis* are reported herein for the first time.

Other plastisphere bacteria, including *Enterobacter*, *Nitratireductor*, *Pelagibacterium*, *Hyphomonas*, and *Marinobacter*, only constituted a small fraction. *Nitratireductor* and *Marinobacter*, which are deep-sea hydrocarbonoclastic bacteria, are contaminants in *Phormidium* cultures. Similar to *Marinobacter*, *Nitratireductor* has been shown to exhibit robust growth on *n*-hexadecane as its sole carbon source and possesses hydrocarbon-degrading genes, such as alkane monooxygenase (Sun *et al.*, 2018).

### PE biodegradation assessment

#### SEM imaging of the consortium culture

**Fig. 4.**
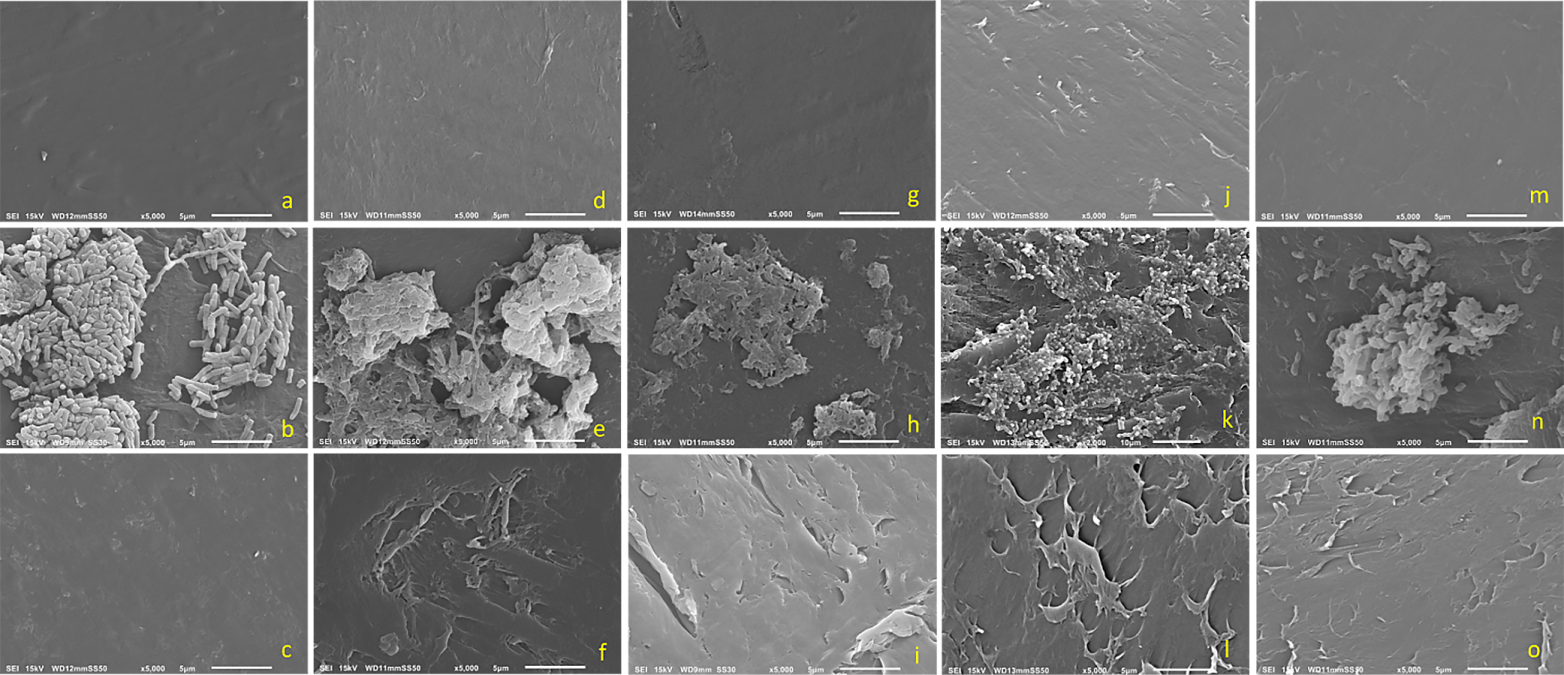
SEM micrographs (a, b, c, d, e, f, g, h, i, j, k, and l: consortium samples). a; Day 10 PE pellet control. b; Day 10 PE pellet biofilm showing multi-layered structures. c; Day 10 PE pellet after biofilm removal. d; Day 60 PE pellet control. e; Day 60 PE pellet showing thick biofilm clusters of consortium bacteria. f; Day 60 PE pellet after biofilm removal showing surface damage. g; Day 120 PE pellet control. h; Day 120 PE pellet showing biofilms. i; Day 120 PE pellet after biofilm removal showing surface damage. j; Day 200 PE pellet control. k; Day 200 PE pellet biofilm showing bacteria embedded in valleys and surface damage. l; Day 200 PE pellet after biofilm removal showing surface damage. (m, n, and o: *Rhodococcus erythropolis* pure culture samples). m; Day 60 PE pellet control. n; Day 60 PE pellet showing *R. erythropolis* biofilms. o; Day 60 PE pellet after *R. erythropolis* biofilm removal showing surface damage.

A SEM ana­lysis of PE samples was performed to assess both bacterial colonization and surface degradation. SEM images obtained from samples on day 10 revealed dense, multilayered biofilms on the PE surface, along with some planktonic cells and no obvious surface damage (Fig. 4a, b, and c). Day 60 PE pellets showed three-dimensional biofilm clusters, in addition to bacteria growing in and accentuating PE pellet surface irregularities. Pits and cavities due to embedded bacteria were noted after biofilm removal (Fig. 4d, e, and f). In later samples (days 120 and 200), bacterial biofilm structures were a mix of cells and debris, bacteria were embedded as pits on the PE surface and grew in the surface crevices of the PE pellet, and irregularities on the PE surface became more prominent at this stage (Fig. 4g, h, i, j, k, and l). After the removal of bacterial biofilms, PE pellets from days 60, 120, and 200 revealed pits and cavities and showed flaking and surface damage on treated PE pellets due to bacterial colonization and degradation. This degradation was attributed to the collective effects of the bacterial consortium community. Additional FTIR ana­lyses further corroborated this biodegradation by revealing the generation of PE oxidative products in the initial days, which were then consumed over time (See Supplemental materials).

#### SEM imaging of a *R. erythropolis* pure culture

The decomposition of the PE surface was confirmed by SEM, and a consortium community ana­lysis showed that *R. erythropolis* was the predominant strain on the PE surface; therefore, this strain was selected and cultured with PE to evaluate its individual colonization and biodegradation ability. Fig. 4m, n, and o show SEM micrographs of day 60 PE pellets incubated with *R. erythropolis* alone. Multilayered biofilms were observed on the PE pellet, similar to the results of the consortium study. Surface erosion was also noted, as shown in Fig. 4f. The appearance of the PE surface treated with *R. erythropolis* was similar to that in the consortium study (Fig. 4o). However, it was not possible to compare erosion efficiency because their initial biomasses were not normalized.

The results of the SEM ana­lysis indicate that *R. erythropolis* exhibited the ability to deteriorate PE and played a major role in the consortium. Although further studies are needed to clarify the individual contribution of other consortium strains, *S. griseus*, which was the only strain that exhibited extracellular laccase activity, appeared to be important in the overall deterioration of PE. In the present study, we observed the potential of *R. erythropolis* to robustly colonize and deteriorate PE, which may be further improved via genetic engineering.

Genetic engineering is another approach to overcome individual bacterial metabolic limitations and involves genetically modifying a suitable host bacterium to express all PE-degrading enzymes heterologously. Through this approach, the regulation of target enzyme expression may be improved and unwanted isozyme expression is less likely, as is often the case in wild-type strains (Arregui *et al.*, 2019).

The heterologous expression of potential PE-degrading enzymes in bacterial/fungal hosts has been demonstrated in the literature. Mo *et al.* reported the expression and secretion of three laccases in *E. coli* (Mo *et al.*, 2022), Whyte *et al.* cloned AH genes from *Rhodococcus* in *E. coli* and *Pseudomonas* (Whyte *et al.*, 2002), and Zadjelovic *et al.* expressed an *Alcanivorax* esterase gene in *E. coli* (Zadjelovic *et al.*, 2020). *E. coli* is commonly used as the bacterial host in most protein expression studies. However, we found that *Rhodococcus* performed better as a plastisphere bacteria than *E. coli*. *Rhodococcus* has been extensively exami­ned for its ability to strongly degrade aromatic and linear hydrocarbons, including polychlorinated biphenyls and dioxins (Kitagawa *et al.*, 2001; Kitagawa *et al.*, 2004). In addition to its hydrocarbon degradative ability, it has also been reported to produce secondary metabolites, such as biosurfactants and antibiotics (Philp *et al.*, 2002; Kitagawa and Tamura, 2008; Inaba *et al.*, 2013).

*Rhodococcus* is easy to culture and grows relatively quickly, and genetic tools, such as plasmid vectors, gene disruption methods, and genome manipulation methods, have been intensively investigated (Kitagawa *et al.*, 2013, Saito *et al.*, 2019; Kitagawa and Hata, 2023), making *Rhodococcus* an excellent host for gene expression. The present results indicate the potential of *R. erythropolis* as a host for genetic engineering studies. Its ability to form robust biofilms on PE surfaces and its genomic potential to extracellularly express beneficial enzymes, such as lipases, esterases, and those involved in alkane utilization, support its suitability for such research.

## Citation

Putcha, J. P., and Kitagawa, W. (2024) Polyethylene Biodegradation by an Artificial Bacterial Consortium: *Rhodococcus* as a Competitive Plastisphere Species. *Microbes Environ ***39**: ME24031.

https://doi.org/10.1264/jsme2.ME24031

## Supplementary Material

Supplementary Material

## Figures and Tables

**Table 1.** Bacterial strains used in the present study

Bacterial strain	Relative growth*	Reference Sequence Accession No.
*Hyphomonas neptunium* NBRC 14232	S	NC_008358.1
*Hyphomonas polymorpha* NBRC 102482	S	ARYM00000000.1
*Brevibacillus borstelensis* NBRC 15714	S	BJOK00000000.1
*Alcanivorax* sp. NBRC 101098	I	AP014613.1
*Alcanivorax profundi* JCM 31866	I	QYYA00000000.1
*Enterobacter asburiae* NBRC 109912	S	CP011863.1
*Bacillus subtilis* JCM 1465T	S	AP019714.1
*Pseudomonas fluorescens* JCM 5963T	I	VFEP00000000.1
*E. coli* K-12	S	NC_000913.3
*Streptomyces griseus* NBRC 13350	S	AP009493.1
*Rhodococcus ruber* JCM 3205T	VF	BCXE00000000.1
*Rhodococcus rhodochrous* JCM 3202T	F	LT906450.1
*Rhodococcus jostii* RHA1	VF	NC_008268.1
*Rhodococcus erythropolis* NBRC 100887	F	AP008957.1
*Rhodococcus zopfii* DSM 44189	F	WBMO00000000.1
*Phormidium *sp. NBRC 102691 (green)	—	NA
*Phormidium *sp. NBRC 102724 (yellow)	—	NA

NBRC: Biological Resource Center, NITE (Japan) JCM: Japan Collection of Microorganisms DSM: Deutsche Sammlung von Mikroorganismen und Zellkulturen GmbH NA: Not available *Relative growth in W medium+0.5% hexadecane. The incubation times required to reach an OD_660_ of 2 were <24 h, <28 h, <30 h, and >30‍ ‍h for VF (very fast), F (fast), I (intermediate), and S (slow), respectively.

**Table 2.** Extracellular enzyme activity and alkane utilization on agar plates

Bacterial strain	Laccase	Esterase	Lipase	Alkane utilization		
				hexadecane	eicosane	dotriacontane
*Hyphomonas neptunium* NBRC 14232	–, (int+)	**+**	**+**	fG	ng	ng
*Hyphomonas polymorpha* NBRC 102482	–, (int+)	**+**	**+**	GR	ng	ng
*Brevibacillus borstelensis* NBRC 15714	–, (int+)	ng	**+**	fG	fG	fG
*Alcanivorax* sp. NBRC 101098	ng	ng	**+**	ng	ng	ng
*Alcanivorax profundi* JCM 31866	ng	ng	**+**	ng	ng	ng
*Enterobacter asburiae* NBRC 109912	–, (int+)	**+**	**+**	GR	fG	fG
*Bacillus subtilis* JCM 1465T	–, (int–)	**+**	**+**	fG	fG	fG
*Pseudomonas fluorescens* JCM 5963T	–, (int+)	**+**	**+**	fG	fG	fG
*E. coli* K-12	–, (int+)	**+**	**+**	fG	fG	fG
*Streptomyces griseus* NBRC 13350	**+**	**+**	**+**	fG	fG	fG
*Rhodococcus ruber* JCM 3205T	–, (int+)	**+**	**+**	GR	GR, CL	GR
*Rhodococcus rhodochrous* JCM 3202T	–, (int+)	**+**	**+**	GR	GR, CL	GR
*Rhodococcus jostii* RHA1	–, (int+)	**+**	**+**	GR	GR, CL	GR
*Rhodococcus erythropolis* NBRC 100887	–, (int+)	**+**	**+**	GR	GR, CL	GR
*Rhodococcus zopfii* DSM 44189	–, (int+)	**+**	**+**	GR	GR, CL	GR

+: extracellular activity positive, –: extracellular activity negative (int+): intracellular activity positive, (int–): intracellular activity negative GR: Growth, ng: no growth, fG: faint growth, CL: a clear zone was observed
