# Supplementary material for: Polyethylene Biodegradation by an Artificial Bacterial Consortium: Rhodococcus as a Competitive Plastisphere Species
Source: Microbes Environ. 2024 Jul 31;39(3):ME24031. doi: 10.1264/jsme2.ME24031 (PMC11427307; doi:10.1264/jsme2.ME24031)
Supplement: Supplementary file 1 — Supplementary Material [file 39_24031_s1.pdf]

**Supplemental Material**

# Polyethylene biodegradation by an artificial bacterial consortium: *Rhodococcus* as a competitive plastisphere species

By Putcha Jyothi Priya and Wataru Kitagawa

**Contents**

1. **Table S1**
   Characteristics of reference stains from literature

2. **Figure S1**
   Pie charts of Day 120 consortium community in the culture broth vs. plastisphere.

3. **FTIR analysis Data in detail**

4. **Figure S2**
   FTIR spectra of Day 25 and Day 200 200 PE treatment samples and their respective controls. Regions shown: (a)1300-800, (b)1680-1600, (c)3600-3100.

5. **References**

## Supplemental Material 1

### Table S1. Characteristics of reference stains from literature.

| Reference strains from literature | Substrate | References | Relation to the strain used in this study |
|---|---|---|---|
| *Hyphomonas* | Plastic marine debris bacteria | (Zettler *et al.* 2013) | Same genera |
| *Brevibacillus borstelensis* strain 707 | LDPE degradation | (Hadad *et al.* 2005) | Same species |
| *Alcanivorax borkumensis* | LDPE degradation | (Delacuvellerie *et al.* 2019) | Same genera |
| *Enterobacter asburiae* YT1 | LDPE degradation | (Yang *et al.* 2014) | Same species |
| *Bacillus subtilis* ATCC 6051 | LDPE degradation | (Yao *et al.* 2022) | Same strain (JCM 1465T = ATCC 6051) |
| *Pseudomonas fluorescens* ATCC 13525 | LDPE degradation | (Mukherjee *et al.* 2018) | Same strain (JCM 5963T = ATCC 13525) |
| *E. coli* K-12 | Biofilm formation on PE and other plastics | (Ganesan *et al.* 2022) | Same strain |
| *Streptomyces sp.* | PE (containing 6% starch) degradation | (El-Shafei *et al.* 1998) | Same genera |
| *Rhodococcus ruber* C208* | LDPE degradation | (Gilan *et al.* 2004) | Same species |
| *Rhodococcus rhodochrous* ATCC 13808* | Plasticizer degradation, | (Sauvageau *et al.* 2009) | Same strain (JCM 3202T = ATCC 13808) |
| *Rhodococcus rhodochrous* ATCC 29672* | PE (containing TDPA) degradation | (Bonhomme *et al.* 2003) | Same species |
| *Rhodococcus jostii* RHA1* | *n*-alkane degradation | (Gibu *et al.* 2019) | Same strain |
| *Rhodococcus erythropolis* PR4 NBRC 100887* | Hydrocarbon degradation | (Laczi *et al.* 2015) | Same strain |
| *Rhodococcus zopfii** | Crude oil degradation | (Pi *et al.* 2017) | Same species |
| *Phormidium lucidum* | LDPE degradation | (Sarmah & Rout 2018) | Same genera |

**Rhodococcus* plastic-degrading enzymes genomic study (Zampolli *et al*. 2022)

## Supplemental Material 2

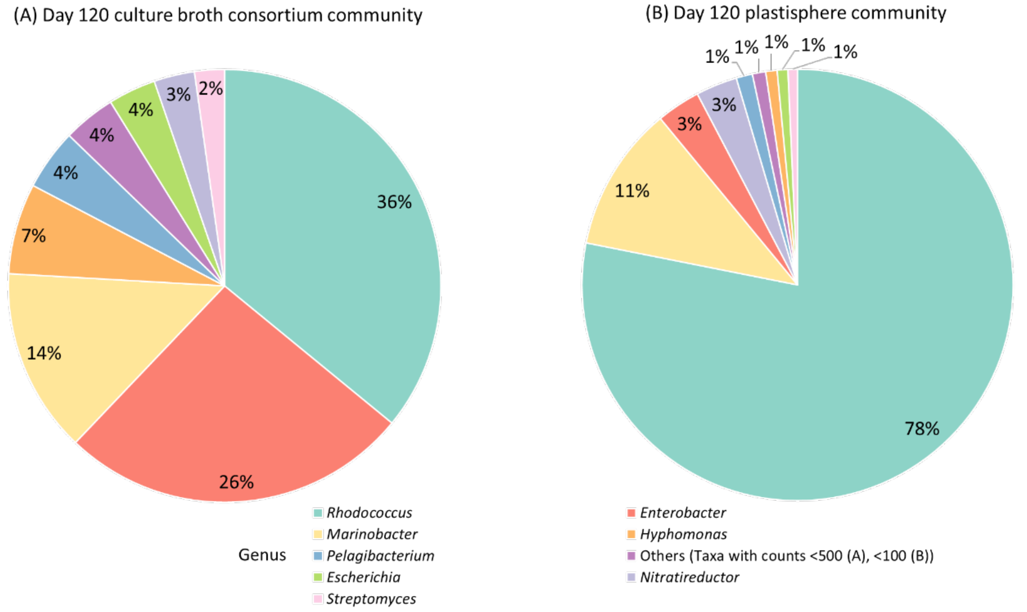


**Figure S1.** Pie charts of Day 120 consortium community in the culture broth vs. plastisphere

## Supplemental Material 3

### Fourier transform infrared spectroscopy (FTIR) analysis Data in detail

### Materials and Methods

For FTIR analysis, the PE samples were collected after biofilm removal, and the respective control samples were also collected. For the biofilm removal, the PE samples were treated with proteinase K (final concentration, 1 mg/mL) then with 2% SDS for 3–4 hours at 37°C. Afterward, the samples were washed thrice with warm, sterile distilled water, followed by a 15-min wash in 70% ethanol. FTIR spectra were obtained in the transmission mode using an ATR PRO ONE FT/IR-6600 (JASCO). Spectra were recorded at a resolution of 4 $cm^{-1}$ in the wavenumber range of 4000–400 $cm^{-1}$. (The FTIR analysis was carried out at the Chitose Institute of Science and Technology, Advanced Research Infrastructure for Materials and Nanotechnology of Japan).

### Results

It has been previously reported that, upon reaction with microbial exoenzymes (such as laccases and AHs), the PE polymer can be broken down into smaller oligomers, which are taken up by bacterial cells where they are further metabolized via the β-oxidation pathway and mineralized to $CO_2$ and $H_2O$ (Ghatge *et al.* 2020, Tao *et al.* 2023). Moreover, the microbe-mediated hydroxylation of *n*-alkanes produces their corresponding alcohols, which are then oxidized to aldehydes, ketones, and carboxylic acids (Eubeler *et al.* 2010).

The PE samples used in this study were pellets 3–4 mm in diameter, which were difficult to prepare for FTIR analysis. The obtained FTIR spectra were not sufficiently strong, although several signals supported the biodegradation of PE.

FTIR peaks shows the creation of PE oxidation products by the bacterial enzymes in the initial stages. Day 25 PE treatment samples show peaks corresponding to carbonyl groups (-C=O) with a strong peak at 1647 $cm^{-1}$; also seen are terminal double bonds in the region of 915-905 $cm^{-1}$ with a peak at 908 $cm^{-1}$ and at 842 $cm^{-1}$ (Figure S2). The appearance of carbonyl groups and terminal double bonds (915-905 $cm^{-1}$) have been reported to be a result of biotical degradation of PE (Adithama *et al.* 2023, Albertsson *et al.* 1987, Zhang *et al.* 2020). Also observed are peaks corresponding to O-H stretching (hydroperoxide

and alcohol) at 3182 $cm^{-1}$ and 3394 $cm^{-1}$ in Day 25 PE treatment samples (Figure S2) (Joshi *et al.* 2022, Li *et al.* 2020, Yang *et al.* 2022). Peaks in the 1100 – 1400 $cm^{-1}$ region corresponding to ethers and other –C-O-C groups are also seen in Day 25 PE treatment samples at 1120 $cm^{-1}$,1216 $cm^{-1}$ and 1245 $cm^{-1}$ (Figure S2) (Biki *et al.* 2021, Hou *et al.* 2022, Kowalczyk *et al.* 2016). Additionally, peaks at 1080, 1081 $cm^{-1}$ are seen in Day 25 and Day 200 PE treatment samples, respectively, which correspond to ethers (Biki *et al.* 2021). Peaks observed at Day 25 (842 $cm^{-1}$, 908 $cm^{-1}$,1120 $cm^{-1}$, 1216 $cm^{-1}$, 1245 $cm^{-1}$, 1647 $cm^{-1}$, 3182 $cm^{-1}$ and 3394 $cm^{-1}$) have disappeared or shrunk in the later part (Day 200). These PE degradation products (carbonyls, ethers, hydroperoxides and other alcohols) brought about by the bacterial enzymes, being short-chained and amenable for further enzymatic breakdown, are consumed and hence disappear in the later stages.

## Supplemental Material 4

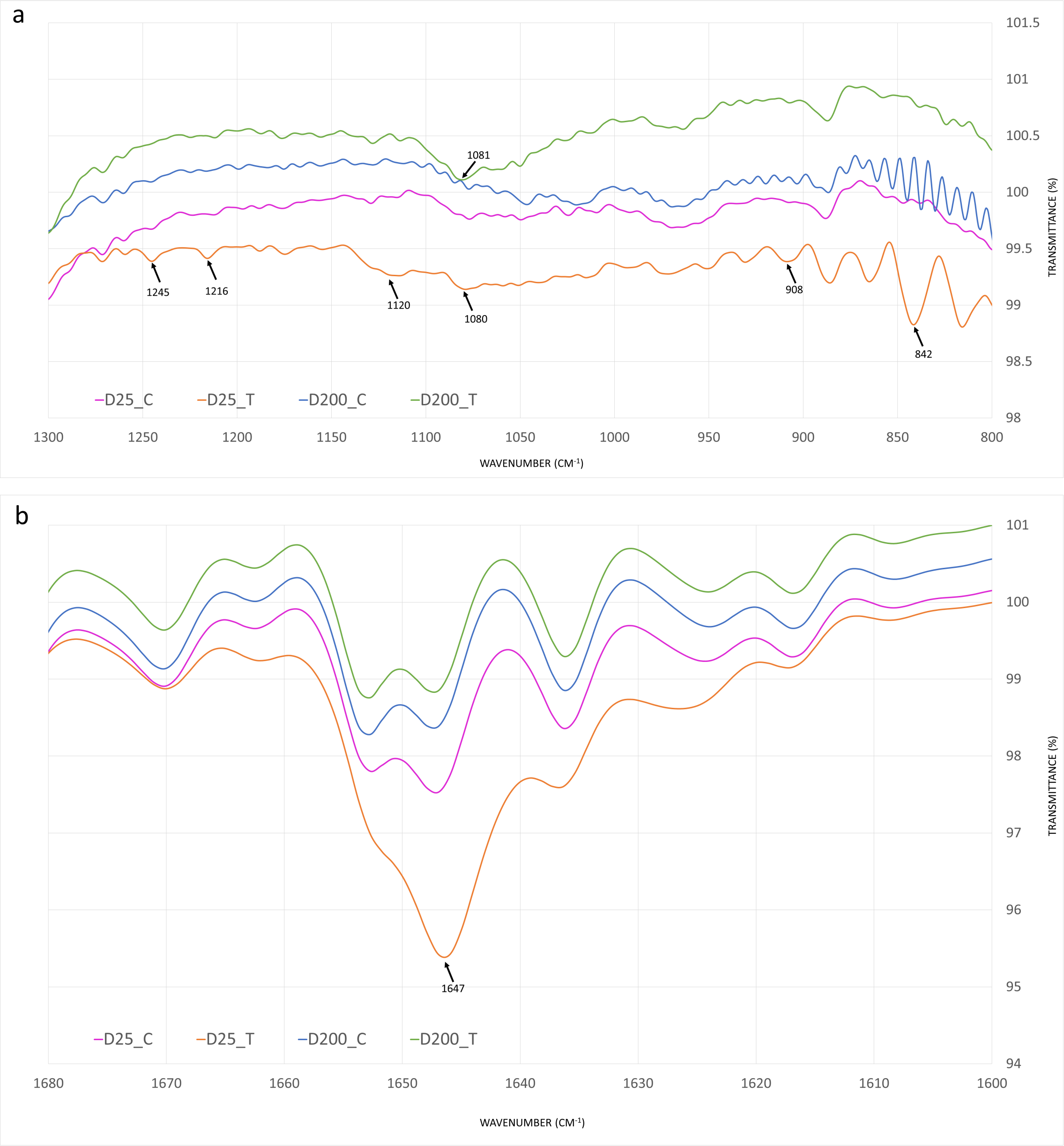


**Figure S2**

FTIR spectra of Day 25 and Day 200 PE treatment samples and their respective controls. Regions shown: (a)1300-800, (b)1680-1600, (c)3600-3100.

## Supplemental Material 4 *continued*

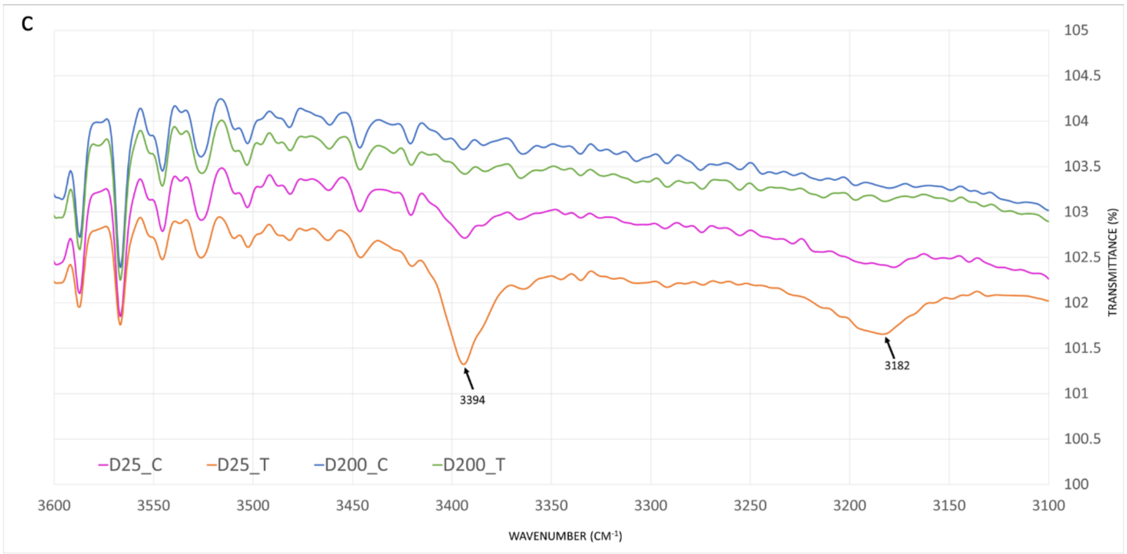


**Figure S2**

FTIR spectra of Day 25 and Day 200 PE treatment samples and their respective controls. Regions shown: (a)1300-800, (b)1680-1600, (c)3600-3100.

## REFERENCES


Adithama, R. M., Munifah, I., Yanto, D. H. Y., and Meryandini, A. (2023) Biodegradation of low-density polyethylene microplastic by new halotolerant bacteria isolated from saline mud in Bledug Kuwu, Indonesia. *Bioresource Technology Reports* **22**: 101466

Albertsson, A. C., Andersson, S. O., and Karlsson, S. (1987) The Mechanism of Biodegradation of Polyethylene. *Polym Degrad Stab* **18**: 73-87

Biki, S. P., Mahmud, S., Akhter, S., Rahman, M. J., Rix, J. J., Al Bachchu, M. A., and Ahmed, M. (2021) Polyethylene degradation by *Ralstonia* sp. strain SKM2 and *Bacillus* sp. strain SM1 isolated from land fill soil site. *Environmental Technology & Innovation* **22**: 101495

Bonhomme, S., Cuer, A., Delort, A. M., Lemaire, J., Sancelme, M., and Scott, G. (2003) Environmental biodegradation of polyethylene. *Polym Degrad Stab* **81**: 441-452

Delacuvellerie, A., Cyriaque, V., Gobert, S., Benali, S., and Wattiez, R. (2019) The plastisphere in marine ecosystem hosts potential specific microbial degraders including *Alcanivorax borkumensis* as a key player for the low-density polyethylene degradation. *J Hazard Mater* **380**: 120899

El-Shafei, H. A., Abd El-Nasser, N. H., Kansoh, A. L., and Ali, A. M. (1998) Biodegradation of disposable polyethylene by fungi and *Streptomyces* species. *Polym Degrad Stab* **62**: 361-365

Eubeler, J. P., Bernhard, M., and Knepper, T. P. (2010) Environmental biodegradation of synthetic polymers II. Biodegradation of different polymer groups. *Trac-Trends in Analytical Chemistry* **29**: 84-100

Ganesan, S., Ruendee, T., Kimura, S. Y., Chawengkijwanich, C., and Janjaroen, D. (2022) Effect of biofilm formation on different types of plastic shopping bags: Structural and physicochemical properties. *Environ Res* **206**: 112542

Ghatge, S., Yang, Y., Ahn, J. H., and Hur, H. G. (2020) Biodegradation of polyethylene: a brief review. *Applied Biological Chemistry* **63**: 27

Gibu, N., Kasai, D., Ikawa, T., Akiyama, E., and Fukuda, M. (2019) Characterization and transcriptional regulation of *n*-alkane hydroxylase gene cluster of *Rhodococcus jostii* RHA1. *Microorganisms* **7**: 479

Gilan, I., Hadar, Y., and Sivan, A. (2004) Colonization, biofilm formation and biodegradation of polyethylene by a strain of *Rhodococcus ruber*. *Appl Microbiol Biotechnol* **65**: 97-104

Hadad, D., Geresh, S., and Sivan, A. (2005) Biodegradation of polyethylene by the thermophilic bacterium *Brevibacillus borstelensis*. *J Appl Microbiol* **98**: 1093-1100

Hou, L. J., Xi, J., Liu, J. X., Wang, P. Y., Xu, T. Q., Liu, T. T.*, et al.* (2022) Biodegradability of polyethylene mulching film by two *Pseudomonas* bacteria and their potential degradation mechanism. *Chemosphere* **286**: 131758

Joshi, G., Goswami, P., Verma, P., Prakash, G., Simon, P., Vinithkumar, N. V., and Dharani, G. (2022) Unraveling the plastic degradation potentials of the plastisphere-associated marine bacterial consortium as a key player for the low-density polyethylene degradation. *J Hazard Mater* **425**: 128005

Kowalczyk, A., Chyc, M., Ryszka, P., and Latowski, D. (2016) *Achromobacter xylosoxidans* as a new microorganism strain colonizing high-density polyethylene as a key step to its biodegradation. *Environmental Science and Pollution Research* **23**: 11349-11356

Laczi, K., Kis, A., Horváth, B., Maróti, G., Hegedüs, B., Perei, K., and Rákhely, G. (2015) Metabolic responses of *Rhodococcus erythropolis* PR4 grown on diesel oil and various hydrocarbons. *Appl Microbiol Biotechnol* **99**: 9745-9759

Li, Z. Y., Wei, R., Gao, M. X., Ren, Y. R., Yu, B., Nie, K. L., *et al.* (2020) Biodegradation of low-density polyethylene by *Microbulbifer hydrolyticus* IRE-31. *Journal of Environmental Management* **263**: 110402

Mukherjee, S., RoyChaudhuri, U., and Kundu, P. P. (2018) Biodegradation of polyethylene via complete solubilization by the action of *Pseudomonas fluorescens*, biosurfactant produced by *Bacillus licheniformis* and anionic surfactant. *Journal of Chemical Technology & Biotechnology* **93**: 1300-1311

Pi, Y., Chen, B., Bao, M., Fan, F., Cai, Q., Ze, L., and Zhang, B. (2017) Microbial degradation of four crude oil by biosurfactant producing strain *Rhodococcus* sp. *Bioresource Technology* **232**: 263-269

Sarmah, P., and Rout, J. (2018) Efficient biodegradation of low-density polyethylene by cyanobacteria isolated from submerged polyethylene surface in domestic sewage water. *Environmental Science and Pollution Research* **25**: 33508-33520

Sauvageau, D., Cooper, D. G., and Nicell, J. A. (2009) Relative rates and mechanisms of biodegradation of diester plasticizers mediated by *Rhodococcus rhodochrous*. *The Canadian Journal of Chemical Engineering* **87**: 499-506

Tao, X. Y., Ouyang, H. R., Zhou, A. F., Wang, D. Y., Matlock, H., Morgan, J. S., *et al.* (2023) Polyethylene Degradation by a *Rhodococcus* Strain Isolated from Naturally Weathered Plastic Waste Enrichment. *Environ Sci Technol* **57**: 13901-13911

Yang, J., Yang, Y., Wu, W. M., Zhao, J., and Jiang, L. (2014) Evidence of Polyethylene Biodegradation by Bacterial Strains from the Guts of Plastic-Eating Waxworms. *Environ Sci Technol* **48**: 13776-13784

Yang, S. S., Ding, M. Q., Ren, X. R., Zhang, Z. R., Li, M. X., Zhang, L. L., *et al.* (2022) Impacts of physical-chemical property of polyethylene on depolymerization and biodegradation in yellow and dark mealworms with high purity microplastics. *Sci Total Environ* **828**: 154458

Yao, Z., Seong, H. J., and Jang, Y.-S. (2022) Degradation of low density polyethylene by *Bacillus* species. *Applied Biological Chemistry* **65**: 84

Zampolli, J., Orro, A., Vezzini, D., and Di Gennaro, P. (2022) Genome-based exploration of *Rhodococcus* species for plastic-degrading genetic determinants using bioinformatic analysis. *Microorganisms* **10**: 1846

Zettler, E. R., Mincer, T. J., and Amaral-Zettler, L. A. (2013) Life in the "Plastisphere": Microbial communities on plastic marine debris. *Environ Sci Technol* **47**: 7137-7146

Zhang, J. Q., Gao, D. L., Li, Q. H., Zhao, Y. X., Li, L., Lin, H. F., *et al.* (2020) Biodegradation of polyethylene microplastic particles by the fungus *Aspergillus flavus* from the guts of wax moth *Galleria mellonella*. *Sci Total Environ* **704**: 135931
